# What do Cochrane Systematic Reviews say about conservative and surgical therapeutic interventions for treating rotator cuff disease? Synthesis of evidence

**DOI:** 10.1590/1516-3180.2019.0275160919

**Published:** 2020-03-06

**Authors:** Eduardo Signorini Bicas Franco, Maria Eduarda dos Santos Puga, Aline Mizusaki Imoto, Jhony de Almeida, Vitor da Mata, Stella Peccin

**Affiliations:** I MSc. Doctoral Student and Physiotherapist, Universidade Federal de São Paulo (UNIFESP), São Paulo (SP), Brazil.; II PhD. Librarian and Professor, Universidade Federal de São Paulo (UNIFESP), São Paulo (SP), Brazil.; III PhD. Physiotherapist and Professor, College of Health Sciences, Brasília (DF), Brazil.; IV PT. Master’s Student and Physiotherapist, Universidade Federal de São Paulo (UNIFESP), Santos (SP), Brazil.; V Physiotherapy Student, Universidade Federal de São Paulo (UNIFESP), Santos (SP), Brazil.; VI PhD. Physiotherapist and Professor, Universidade Federal de São Paulo (UNIFESP), Santos (SP), Brazil.

**Keywords:** Shoulder, Rotator cuff, Exercise, Shoulder joint, Physiotherapy, Labral repair, Shoulder disorder, Conservative shoulder treatment

## Abstract

**BACKGROUND:**

Shoulder pain is considered to be the third largest cause of musculoskeletal functional alterations in individuals presenting pain during movement.

**OBJECTIVE:**

The purpose of this synthesis of evidence was to identify the clinical effectiveness of conservative and surgical treatments reported in Cochrane systematic reviews among individuals diagnosed with rotator cuff disease.

**DESIGNAND SETTING:**

Review of systematic reviews, conducted in the Federal University of São Paulo (Universidade Federal de São Paulo, UNIFESP).

**METHODS:**

This synthesis of evidence included systematic reviews that had been published in the Cochrane database. The inclusion criteria were that these systematic reviews should involve individuals aged ≥ 16 years with rotator cuff disease, comparing surgical procedures with or without associated nonsurgical procedures versus placebo, no treatment or other nonsurgical interventions.

**RESULTS:**

Thirty-one systematic reviews were included, involving comparisons between surgical procedures and conservative treatment; procedures either combined or not combined with drugs, versus other procedures; and procedures involving exercises, manual therapy and electrothermal or phototherapeutic resources.

**CONCLUSIONS:**

The findings suggest that strengthening exercises, with or without associated manual therapy techniques and other resources, were the interventions with greatest power of treatment over the medium and long terms, for individuals with shoulder pain. These had greater therapeutic power than surgical procedures, electrotherapy or photobiomodulation.

Protocol registration number in the PROSPERO database: ID - CRD42018096578.

## INTRODUCTION

Shoulder pain is considered to be the third most important musculoskeletal complaint that leads individuals to seek some type of primary care. Its prevalence in the general population is 7% to 26%. Most complaints of shoulder pain relate to rotator cuff disease, which is responsible for 4.5 million cases per year, attended by healthcare professionals in the United States.^1^^,^^2^

The term “rotator cuff disease” is used to refer to a set of conditions, regardless of cause and specific area of the injury. It may encompass conditions ranging from partial to total ruptures, as well as tendinopathies and tendinosis.^3^^,^^4^ This divergence in the definition of this term is closely related to the diversity of technical terms that have been described and used for such conditions. These have multifactorial causes (mechanical, biological and social factors) and, consequently, considerable influence on patients’ performance.^3^^,^^4^^,^^5^

Rotator cuff injury leads to pain, functional impairment and psychological distress.^6^^,^^7^^,^^8^^,^^9^ These conditions start a cascade of consequences involving increased symptoms and functional incapacity. The possibility of chronic injury caused by lack of ideal treatment for these types of lesions also needs to be considered. Chronicity often leads these individuals into scenarios of exclusion and worsening of the condition, both physically and mentally.^10^^,^^11^

## OBJECTIVE

Thus, the purpose of this synthesis of evidence was to identify the clinical effectiveness of conservative and surgical treatments for individuals diagnosed with rotator cuff disease that are described in Cochrane systematic reviews.

## METHODS

### Design

This synthesis of evidence comprised a summary of systematic reviews that have been published in the Cochrane database. There were no restrictions on the date and language of publication of the studies included in this synthesis.

### Inclusion criteria

#### Types of participants

Individuals aged ≥ 16 years with rotator cuff disease were considered, irrespective of the time of onset of the injury and the symptoms presented. Diagnostic confirmation of these participants’ conditions was clarified in the body of the text. Systematic reviews involving only individuals with painful symptoms or any other symptom in the shoulder complex without diagnostic confirmation of rotator cuff disease were not considered for this synthesis of evidence.

#### Types of interventions

The interventions considered for this synthesis of evidence were the following: surgical procedures with or without associated nonsurgical procedures, compared with placebo, no treatment or other nonsurgical intervention; or nonsurgical procedures with or without associated surgical procedures, compared with placebo, no treatment or other non-surgical or surgical intervention.

The following studies were not included in this review: systematic reviews comparing two or more surgical procedures or techniques for shoulder problems (for example, open versus arthroscopic surgery) and systematic reviews that investigated the effects of revision surgeries on the shoulder complex or prosthesis placement in the glenohumeral joint.

#### Types of outcomes

We considered any outcomes (pain, function, range of motion etc.) that were found in the studies.

#### Types of comparison

The following comparisons regarding the intervention were considered: (1) surgical procedures versus conservative treatment; (2) procedures either combined or not combined with drugs, versus other procedures; and (3) comparisons between procedures involving exercises, manual therapy and electrothermal or phototherapeutic resources.

### Search and study selection process

The search for systematic reviews was conducted between March 30, 2017, and February 3, 2019, by two authors (Franco ESB and Puga MES), using the official medical subject headings (MeSH) terminology, in the Cochrane Library database (via Wiley). The search strategy can be seen in Table 1. Two authors (Franco ESB and Mizusaki Imoto A) selected the studies, respecting the inclusion criteria described above. In cases of disagreement, discussions were held to arrive at a consensus. When this was not possible, the opinion of a third author was requested. Only reviews published in the Cochrane Library were included.

**Table 1.** Search strategy

#1 MeSH descriptor: [Rotator Cuff Injuries] explode all trees
#2 Cuff Injury, Rotator or Injuries, Rotator Cuff or Injury, Rotator Cuff or Rotator Cuff Injury or Rotator Cuff Tears or Rotator Cuff Tear or Tear, Rotator Cuff or Tears, Rotator Cuff or Rotator Cuff Tendinosis or Rotator Cuff Tendinoses or Tendinoses, Rotator Cuff or Tendinosis, Rotator Cuff or Rotator Cuff Tendinitis or Rotator Cuff Tendinitides or Tendinitis, Rotator Cuff or Glenoid Labral Tears or Glenoid Labral Tear or Labral Tear, Glenoid or Labral Tears, Glenoid or Tear, Glenoid Labral or Tears, Glenoid Labral
#3 #1 OR #2

The selection process was carried out in two stages. Firstly, studies were selected according to their title and summary, using the PICOS criteria (population or problem, intervention, comparison, outcome and study design). Secondly, studies were selected from the full text. When the first step was deemed insufficient for the authors to make their decisions, the study was accessed and the analysis was based on the full text.

Data extraction was performed by two evaluators (Franco ESB and Mata V), from the original files of the systematic reviews, using a predetermined digital extraction sheet containing the following main points: year of publication, authors’ names and name of periodical, number of primary studies included in the review, types and numbers of participants, interventions and outcomes, analysis of bias and adjustments made, details of intervention groups, duration and parameters of the study, follow-up period, assessment tools and, when present, statistical values expressed through meta-analysis, relative risk format, differences between standardized and non-standardized means or confidence intervals.

A single author (Franco ESB) used the Review Manager software (RevMan) 5.3 to compile all information extracted from the systematic reviews. For this review, the data were synthetized by the group of researchers involved (Franco ESB, Puga MES and Peccin S), using the RevMan 5.3 software. From the data extracted, subgroups of the systematic reviews with Cochrane methodology were created and a subdivision for each outcome was elaborated.

All the reviews included for the final synthesis incorporated the following features from the systematic reviews: project published a priori; selection and extraction of data from trials performed by two independent evaluators; electronic searches always performed using more than two sources, with search strategies presented in the body of the text; lists containing the primary studies included and excluded, with detailing of the characteristics of the studies included presented in the body of the text; analysis of the methodological quality of the primary studies through evaluation instruments; appropriate methods for combining the results of the primary studies, so as to ensure the homogeneity or heterogeneity of the final product; and conflicts of interest reported by the authors.

The quantitative analyses on continuous variables were grouped in terms of the mean difference (MD) or standardized mean difference (SMD) with the 95% confidence interval (CI). The heterogeneity presented was calculated in terms of I².

This study was approved by the Research Ethics Committee of the Federal University of São Paulo (Universidade Federal de São Paulo, UNIFESP) under the number 4095131216 on January 16, 2017.

## RESULTS

The search strategy found 783 studies in the Cochrane database, among which there were 31 systematic reviews, 9 protocols, 739 primary studies, one editorial and three clinical answers. In compliance with the inclusion criteria, eight studies were considered eligible for further qualitative analysis (Table 2).^1^^,^^12^^,^^13^^,^^14^^,^^15^^,^^16^^,^^17^^,^^18^

**Table 2.** Characteristics of the interventions and main findings from comparisons between them, seen in Cochrane systematic reviews in relation to the target population (i.e. individuals with rotator cuff disease)

Comparison	Results	Quality of evidence (GRADE)
Subacromial decompression versus conservative treatment	After 12 months of follow-up, there were no differences between the groups, in evaluations at 3, 6 and 12 months (outcome: function)	Not described
Corticosteroid versus placebo	Reduction of pain and improved function seen over the short term, favoring the group that used the drug.	Not described
Subacromial drugs guided via US versus undirected subacromial drugs	Pain reduction (after 6 weeks) and short-term active ROM improvement (after 1 and 2 weeks), favoring the group that used the guide	Moderate (pain after 6 weeks) Not described (active ROM)
Glycerin trinitrate versus placebo	Reduction favoring intervention within 24-48 hours after application	Low
Manual therapy + exercise versus exercise	Improvement of the pain and function symptoms for the group with manual therapy + exercise after 3 and 4 weeks	Not described
LLLT versus placebo	Reduction of pain and improvement of ROM after 2 and 3 weeks, favoring the LLLT group	Low (pain and active ROM after 2 and 3 weeks)
LLLT versus non-steroidal anti-inflammatory drugs	Reduction of pain after 2 weeks, favoring the LLLT group	Very low
TENS versus placebo	Reduction of pain after application, favoring the TENS group	Very low
Glucocorticoid + exercise versus TENS + exercise	Reduction of pain and improvement of function over the short term (1 week), favoring the group with drug + exercise	Not described
Naturopathy + medication versus exercise + manual therapy	Reduction of pain and improvement of function, favoring use of the naturopathic method, after 12 weeks	Low
*Diacutaneous fibrillation versus placebo	Pain reduction (immediately) and active ROM improvement, favoring the intervention group shortly after the procedure	Low
Massage versus no treatment	Reduction of pain and improvement of function, favoring the intervention group, after 2 weeks	Very low
FNP versus US	Pain reduction, favoring the intervention group shortly after the procedure (immediately)	Not described
Exercise versus subacromial decompression	After 12 months of follow-up, there were no differences between the groups, in evaluations at 6 and 12 months (outcome: pain and function)	Low (pain after 6 months) Low (function after 6 months)
Training based on exercise versus no treatment	Reduction of pain and improvement of function, favoring the intervention group, after 8 and 10 weeks	Very low (pain and function)
Specific exercises versus nonspecific exercises	Reduction of pain and improvement of function, favoring the specific group, after 3 months	High (pain and function after 3 months)
“Jing Luo” acupuncture versus traditional acupuncture	Improvement of the recovery level, favoring the “Jing Luo” method over the short term	Not described

*Diacutaneous fibrillation: a technique known as crocheting, in which therapeutic “hooks” are used.

PNF = proprioceptive neuromuscular facilitation; RCD = rotator cuff disease; ROM = range of motion; US = ultrasound; TENS = transcutaneous electrical nerve stimulation; LLLT = low-level laser therapy.

### 1) Surgical procedures versus conservative treatment

#### Pain

One systematic review investigated comparisons between surgical and conservative treatments among 90 participants with a mean age of 44 years. The surgical procedure comprised subacromial decompression (bursectomy with partial resection of the anteroinferior extremity of the acromion and the coracoacromial ligament) via an arthroscopic approach, followed by a rehabilitation process composed of physiotherapy and exercise. The conservative treatment comprised a process of 19 visits over a 12-week period. No differences between the groups were observed in evaluations at the 3^rd^, 6^th^ and 12^th^ months after these treatments (MD -4.6, 95% CI -12.48 to 3.28; MD -1.4, 95% CI -10.43 to 7.63; and MD -4.5, 95% CI -13.73 to 4.73, respectively).^12^

#### Treatment success

Comparative analysis was done on the open-ended acromioplasty followed by a physiotherapy process that started three months after surgery, in relation to the conservative treatment, which comprised exercises and guidance for the participants identified in that review.^12^ At the end of the 6^th^ and 12^th^ months, there were no significant differences between the groups regarding treatment success, defined as reduction of pain symptoms by more than 50%. After six months, the relative risk (RR) was 1.07 (95% CI 0.34 to 3.4); 5 patients out of a total of 21 (surgical group) and 4 patients out of a total of 18 (non-surgical group) were evaluated. After 12 months, the RR was 1.89 (95% CI 0.81 to 4.41); 11 patients out of a total of 21 (surgical group) and 5 patients out of a total of 18 (non-surgical group) were evaluated.

### 2) Procedures either combined or not combined with drugs versus other procedures

We found five different systematic reviews addressing this subject, from which we could extract data.

#### Pain and range of motion

One review^13^ addressed application of corticosteroids subacromially, compared with placebo, among 160 subjects. A small benefit was found regarding pain relief, measured four weeks after the intervention, compared with the placebo group (SMD 0.83; 95% CI 0.39 to 1.26); and regarding function, measured after the intervention (SMD 0.63; 95% CI 0.20 to 1.06).

Use of different types of drug application was assessed in another review,^14^ in which data were gathered from three studies on 207 participants that compared the effect of ultrasound-guided subacromial application of drugs with the effect of application of these drugs without the presence of a guiding device. Based on the data from the three studies together, pain improvement was observed six weeks after drug application in the group in which the injection was guided (SMD -0.80; 95% CI -1.46 to -0.14). However, these studies presented a high degree of heterogeneity (I² = 79%).

In the same review,^14^ it was found that 40 participants who received ultrasound-guided subacromial application of drugs presented significant improvement in measurements of active abduction between one and two weeks after the application, compared with unguided application (MD 39.29; 95% CI 27.40 to 51.18).^14^

In a third review,^15^ the effect of application of topical glycerin trinitrate at a dosage of 5 mg per day was investigated in comparison with placebo for pain (after 24 hours: SMD -1.05; 95% CI -1.52 to -0.0%), and in the treatment group, 58 patients (after 48 hours: SMD -3.50; 95% CI -3.96 to -3.04), compared with the placebo group. After 15 days, the intervention group presented more participants without any symptoms (RR 1.91; 95% CI 1.04 to 3.50), although the control group suffered a loss of 50% of the participants.^15^

In a fourth review,^1^ glucocorticoid use in association with physical exercise was compared with use of transcutaneous electrical nerve stimulation (TENS) in association with physical exercise. There were 40 participants. It was seen that the results favored use of glucocorticoid in association with home exercises, evaluated after one week (MD 2.10; 95% CI 0.92 to 3.28). Thus, after one week of treatment, the criterion of “treatment success” (number of participants indicating improvement) favored the group that used the drug: TENS group 20% (4/20) versus glucocorticoid group 70% (14/20); RR 0.29; 95% CI 0.11 to 0.72.

Also in this fourth review,^1^ use of a naturopathic intervention in association with acupuncture plus the drug phlogenzym was compared with use of physical exercises in association with manual therapy, among 85 participants. It was shown that, after 12 weeks, there were significant differences that favored the naturopathic group regarding pain (MD 1.30; 95% CI 0.56 to 2.04; versus MD 20.94; 95% CI 6.40 to 35.48).

In a fifth review,^16^ favorable effects regarding pain reduction through application of low-intensity laser were observed in comparison with use of non-steroidal anti-inflammatory drugs after two weeks (MD 2; 95% CI 1.00 to 3.50) in a sample of 40 patients. Another outcome was that the low-intensity laser resulted in some improvement compared with the non-steroidal anti-inflammatory drug group, regarding active shoulder abduction (MD 20 degrees of range of motion, 95% CI 10.00 to 40.00); shoulder flexion (MD 14.99 degrees, 95% CI 5.00 to 29.00); and extension (MD 6 degrees, 95% CI 0.00 to 20.00).

### 3) Comparison between procedures involving exercises, manual therapy and electrothermal and phototherapeutic resources

One review^17^ assessed whether inclusion of a manual therapy program for physical exercise would generate greater benefits in relation to pain, compared with physical exercise alone, in two non-comparable studies. It was found that, after three and four weeks, mobilization performed in association with an exercise program gave rise to greater effects than were seen through exercise alone (MD -186.23, 95% CI -319.34 to -53.12; versus MD -32.07, 95% CI -58.04 to -6.10).^17^

In the same review,^17^ it was shown that application of low-intensity laser in the intervention group was favorable in relation to pain, compared with the placebo group, after two and three weeks of application seen in the primary studies: after two weeks: MD 2.5, 95% CI 2.01 to 3.00; after three weeks: 83% (10/12) versus 42% (5/12); RR 2.00, 95% CI 0.98 to 4.09. Moreover, there was improvement of the active ranges of motion of abduction, flexion and extension, measured in degrees, respectively: MD 20°, 95% CI 10.00 to 40.00; MD 15°, 95% CI 5.00 to 29.00; and MD 6°, 95% CI 0.00 to 20.00.^17^

In another review,^16^ application of electrical currents for pain control was assessed. This review, with 20 participants, showed that application of TENS gave rise to better results shortly after the intervention, compared with placebo. In the intervention group, the mean was 34.8 (ranging from 12 to 68 points on a 100-point scale); and in the control group, the mean was 64.5 (ranging from 38 to 95 points on a 100-point scale).

Another review^1^ involved 50 participants, and it analyzed the use of diacutaneous fibrillation by means of hooks (crocheting) in comparison with placebo (same material, but done superficially) gave rise to a significant difference between the groups. Pain reduction was favored in the intervention group after a single treatment session of approximately 15 minutes (RR 2.14; 95% CI 1.06 to 4.34). These improvements (expressed in degrees) were observed in relation to active abduction (MD 7.30°, 95% CI 2.22° to 12.38°), active flexion (MD 11.40°, 95% CI 5.86° to 16.94°), active extension (MD 1.9°, 95% CI -1.46° to 5.26°) and active medial rotation (MD 3.10°, 95% CI 0.17° to 6.03°).^1^

In the same review,^1^ use of therapeutic massage was compared with a group without any type of treatment, among 29 participants. The massage was applied for 15 to 20 minutes and was administered six times over a two-week period. An evaluation after this two-week period showed that the massage had beneficial effects, compared with the group that did not receive any intervention, in relation to pain (MD -22.00, 95% CI -41.19 to -2.81) and function (MD 7.20, 95% CI 2.20 to 12.20).

The same review^1^ evaluated other interventions: the use of mobilization in association with the proprioceptive neuromuscular facilitation technique was compared with use of therapeutic ultrasound. Among the 30 participants in this review, use of mobilization in association with proprioceptive neuromuscular facilitation was shown to have a positive effect regarding pain reduction immediately after the intervention, in comparison with the other group (MD -1.43, 95% CI -1.97 to -0.89).

The review^1^ also made a comparison between a training group and a non-training group, considering 120 participants. Favorable results regarding pain reduction were found in the training group that performed strengthening exercises, compared with the control group (non-training group), after 8 weeks (MD -1.90, 95% CI -3.27 to -0.53) and 10 weeks (MD -1.30, 95% CI -2.10 to -0.50); and regarding function after 8 weeks (MD -15.50, 95% CI -28.94 to -2.06) and after 10 weeks (MD 6.90, 95% CI 0.59 to 13.21).

Again in the same review¹, use of specific exercises for treating tendinopathies of rotator cuff structures was compared with a nonspecific exercise program, among 97 participants evaluated on a 100-point scale regarding two outcomes: pain and function. After three months, there was a significant difference favoring the specific exercise group in relation to three types of pain: general pain (MD -10.00, 95% CI -18.18 to -1.82), night pain (MD -12.00, 95% CI -21.87 to -2.13) and pain on motion (MD -16.00, 95% CI -26.57 to -5.4); and in relation to function (MD 20.00, 95% CI 11.55 to 28.45). The group that did specific exercises had better results, characterized by a decrease in the symptoms (RR 2.87, 95% CI 1.66 to 4.96) and a smaller number of patients who needed to undergo surgery, 3 and 12 months after the treatment started (RR 0.37, 95% CI 0.22 to 0.64).

The same review^1^ also compared the use of physical exercise in association with manual therapy, with non-treatment. Among the 85 participants in this review, favorable results regarding improved function were found in the intervention group six months after the therapy (MD 19.35).

Supervised exercises were also compared with two interventions:^1^ 1) use of arthroscopic subacromial decompression; and 2) use of low-intensity laser in placebo (off) format. This analysis involved 125 participants. At the end of six months, favorable results were found in the supervised exercise group, compared with the other groups, in relation to pain (MD 10, on a 35-point scale) and in relation to function (MD 10, on a 30-point scale).

In another review,^18^ use of acupuncture was analyzed among 98 participants through comparison of the distribution of points between the “Jing Luo” method and the traditional method. The results showed that there was significant improvement in the recovery level through the Jing Luo method, compared with application of traditional acupuncture (RR 1.50, 95% CI 1.08 to 2.09).

## DISCUSSION

Using the inclusion criteria initially described, eight systematic reviews were considered eligible for this synthesis. All of these reviews included primary studies that involved participants presenting either rotator cuff disease or nonspecific shoulder joint pain. As a form of standardization, only the studies in which there was diagnostic confirmation of rotator cuff dysfunctions were included for the final synthesis. There were 34 primary studies that, following the analysis in the reviews, showed some kind of statistically significant benefit in comparisons between two groups of interventions, but in which the methodological quality was uncertain and, in many cases, was not discussed by the authors of the systematic reviews.

In comparing the wide range of interventions involving subjects with rotator cuff disease, the treatments used some years ago seem to be divergent from what was used more recently. Recent studies have shown scenarios that are more favorable for use of conservative treatments instead of surgical treatment. Invasive procedures such as acromioplasty in association with soft tissue resection have not shown any benefit for patients in terms of pain levels and functionality over the short, medium and long terms. Thus, in keeping with the most recent clinical guidelines, conservative treatment, based mainly on therapeutic exercises either combined or not combined with electrothermal therapeutic devices, has been shown to be more efficient for treating rotator cuff disease.^19^

The risk of bias in the primary studies that was ascertained in the present review was closely linked with the low numbers of participants in many of these studies. It was also especially linked with the short follow-up periods of many interventions, which were often only evaluated over periods of between 24 hours and six weeks.

Standardization of samples of participants such that these subjects all present the same condition (rotator cuff dysfunction in the present study) and definition of post-treatment evaluation periods may enable syntheses involving larger numbers of studies with high degrees of homogeneity and, consequently, higher methodological quality. This will facilitate completion of systematic reviews, since the numbers of homogeneous studies will be higher.

## CONCLUSION

The present review identified eight Cochrane systematic reviews that had assessed conservative and surgical treatments for rotator cuff dysfunctions. The findings suggested that strengthening exercises with or without associated techniques for manual therapy and use of electrothermal or phototherapeutic resources were the interventions with greatest power of treatment for individuals with this condition, over the medium and long terms. These approaches had greater therapeutic power than surgical procedures, which had previously been considered to be the standard treatment for many patients.

## References

[1] Page MJ, Green S, McBain B (2016). Manual therapy and exercise for rotator cuff disease. Cochrane Database Syst Rev.

[2] Mitchell C, Adebajo A, Hay E, Carr A (2005). Shoulder pain: diagnosis and management in primary care. BMJ.

[3] Schellingerhout JM, Verhagen AP, Thomas S, Koes BW (2008). Lack of uniformity in diagnostic labeling of shoulder pain: time for a different approach. Man Ther.

[4] Celik D, Akyuz G, Yeldan I (2009). Comparison of the effects of two different exercise programs on pain in subacromial impingement syndrome. Acta Orthop Traumatol Turc.

[5] Hermans J, Luime JJ, Meuffels DE (2013). Does this patient with shoulder pain have rotator cuff disease? The Rational Clinical Examination systematic review. JAMA.

[6] Jain NB, Wilcox III RB, Katz JN, Higgins LD (2013). Clinical examination of the rotator cuff. PM R.

[7] Bennell K, Coburn S, Wee E (2007). Efficacy and cost-effectiveness of a physiotherapy program for chronic rotator cuff pathology: a protocol for a randomised, double-blind, placebo-controlled trial. BMC Musculoskelet Disord.

[8] Bennell K, Wee E, Coburn S (2010). Efficacy of standardised manual therapy and home exercise programme for chronic rotator cuff disease: randomised placebo controlled trial. BMJ.

[9] Rhon DI, Boyles RB, Cleland JA (2014). One-year outcome of subacromial corticosteroid injection compared with manual physical therapy for the management of the unilateral shoulder impingement syndrome: a pragmatic randomized trial. Ann Intern Med.

[10] Seida JC, LeBlanc C, Schouten JR (2010). Systematic review: nonoperative and operative treatments for rotator cuff tears. Ann Intern Med.

[11] Yamamoto A, Takagishi K, Osawa T (2010). Prevalence and risk factors of a rotator cuff tear in the general population. J Shoulder Elbow Surg.

[12] Coghlan JA, Buchbinder R, Green S, Johnston RV, Bell SN (2008). Surgery for rotator cuff disease. Cochrane Database Syst Rev.

[13] Buchbinder R, Green S, Youd JM (2003). Corticosteroid injections for shoulder pain. Cochrane Database Syst Rev.

[14] Bloom JE, Rischin A, Johnston RV, Buchbinder R (2012). Image‐guided versus blind glucocorticoid injection for shoulder pain. Cochrane Database Syst Rev.

[15] Cumpston M, Johnston RV, Wengier L, Buchbinder R (2009). Topical glyceryl trinitrate for rotator cuff disease. Cochrane Database Syst Rev.

[16] Page MJ, Green S, Mrocki MA (2016). Electrotherapy modalities for rotator cuff disease. Cochrane Database Syst Rev.

[17] Green S, Buchbinder R, Hetrick S (2003). Physiotherapy interventions for shoulder pain. Cochrane Database Syst Rev.

[18] Green S, Buchbinder R, Hetrick S (2005). Acupuncture for shoulder pain. Cochrane Database Syst Rev.

[19] Vandvik PO, Lähdeoja T, Ardern C (2019). Subacromial decompression surgery for adults with shoulder pain: a clinical practice guideline. BMJ.

